# 

**Published:** 1917-07

**Authors:** 


### INDEX FOR VOLUME 72

### AUGUST, 1916, to JULY, 1917

| Page | | Page | |
|---|---|---|---|
| 1— 54 | August | 319—366 | February |
| 55—108 | September | 367—414 | March |
| 109—162 | October | 415—454 | April |
| 163—216 | November | 455—494 | May |
| 217—270 | December | 495—534 | June |
| 271—318 | January | 535—582 | July |

### CONTRIBUTORS:

AARON, Chas. D., Detroit, Chronic Intestinal Stasis from the View-point of the Internist, Aug., page 1.

BARNES, Rollin H., St. Louis, Haemorrhoids and the General Surgeon, Aug., page 11.

BARROWS, Franklin W., Buffalo, Address of President of Medical Society of the County of Erie, N. Y., March, page 374.

BASSLER, Anthony, N. Y., Anaemia (Symposium) June, page 507.

BATESON, S. W., Buffalo, Grading of Milk Supplies, Dec., page 239.

BENEDICT, A. L., Buffalo, Highways, Address of President of Central N. Y. Medical Assn., Jan., page 278.

BETHUNE, Chas. W., Buffalo, Report of Case of Syphilis Cured by one Large Dose of Salvarson, with Reinfection Two Years Later, Oct., page 113.

BONNAR, John D., Buffalo, Compulsory Health Insurance Bill, May, page 472.

BOSTON, L. Napoleon, Philadelphia, Anaemia (Symposium) June, page 507.

CANNON, W. B., New Haven, Curare (Symposium), Sept., page 104.

CLARK, Edward, Buffalo, Poliomyelitis, Dec., page 230.

COLEGROVE, La Rue, Elmira, Determination of Carbon Dioxid Tension of Alveolar Air and Hydrogen-ion Concentration of Blood and Urine, Mch., page 367.

CRILE, Geo. W., Cleveland, Methods and Results in Surgery of the Stomach and Intestine, Sept., page 55.

CROTHERS, T. D., Hartford, Scientific Researches Into the Causes of Alcoholism and Inebriety, Sept., page 64.

DALAND, Judson, Philadelphia, Anaemia (Symposium), June, page 506.

DE NIORD, Richard N., Buffalo, Prognostic Value and Clinical Application of Studies in Retention, Feb., page 319.

DUNCAN, Charles H., New York, Autotherapy in Abdominal Infections, July, page 535.

EWERS, Wm. V., Rochester, Samuel Guthrie, Discovery of Chloroform, May, page 459; June, page 502.

FRONCZAK, Francis E., Buffalo, Case of Rabies in a Boy Nine Years Old, Feb., page 337.

GIRALOTO, Horacio, Palmeiro, Brazil, Method of Shrinking Human Heads by Baqueta Indians, July, page 545.

GLOSSER, H. H., Buffalo, With the Seventy-Fourth Regiment on the Texan Frontier, Oct., page 120.

HALLIBURTON, W. D., London, Curare (Symposium), Sept., page 104.

HENNINGTON, Chas. W., Rochester, Application of the So-called New War-time Antiseptics in the Several Branches of Surgery of Civil Life, Nov., page 163.

HENNINGTON, Chas. W., Rochester, Gall Bladder Surgery, Shall we Drain or Remove? Apr., page 415.

HEYD, Chas. Gordon, N. Y., Association of Pancreatitis and Biliary Affections, June, page 495.

KANE, Evan O'Neill, Kane, Pa., Case History of Centenarian—Cancer—Radium, Aug., page 13.

KANE, Evan O'Neill, Kane, Pa., Bullet in Brain Destroying Sight and Hearing — Recovery, Apr., page 455.

LAPP, H. C., N. Tonawanda, Phylacogen Treatment of Rheumatism, Aug., page 66.

LEVYN, Lester I., Buffalo, Roentgen Diagnosis of the Gastro-Intestinal Tract, Jan., page 271.

SERVOSS, George L., Reno, Nevado, Are We Scientific? July, page 540.

SHARP, Edward A., Buffalo, Differential Diagnosis and Treatment of Infantile Paralysis, Dec., page 224.

SNOW, Irving M., Buffalo, Attitude of Family Physician During an Epidemic of Poliomyelitis, Dec., page 217.

SMITH, B. E., Angola, Indefinite Pain, Its Cause as Viewed by the Dowd Phosphatometer, Oct., page 114.

TROTTER, Homer A., Buffalo, Streptococcus Mucosus Capsulatus, and Its Relation to Otology, Nov., page 169.

VON OEFELE, Felix, N. Y., American Balneology, Oct., page 109; Nov., page 173; Dec., page 234; Jan., page 285; Feb., page 329; Mch., page 376; Apr., page 420; May, page 464.

WARBRICK, John C., Chicago, Spurs in the Nose, Sept., page 61.

WARBRICK, John C., Chicago, The Pharynx, Jan., page 327.

**OBITUARY:**

Page

Ainsworth, Elbert Augustus, Dec. ....268
Aldrich, John O., Feb....360
Allen, Duncan S., Jan....315
Appley, Otto, Mch....413

Bailey, Alexander, Mch....413
Baldwin, Evelyn, May....493
Bangs, Edwin Mayo, Feb....358
Baum, Henry Clay, Sept....106
Beacham, Joseph, Nov....211
Bender, Ida C., Aug.... 41
Bennett, Charles E., Feb....360
Bessemer, Martin, Jan....314
Betts, George W., July....569
Bills, Walter H., Aug.... 41
Block, D. Eugene, Jan....315
Brewer, Fitch, July....569
Brunton, T. Lauder, Dec....268
Bryan, Lucius W., Nov....211

Chapman, Eugene Adelbert, Mch. ....414
Clarke, Frank C., Sept....106
Cole, Charles Gray, Jan....315
Cook, Edward J., July....569
Coppernoll, Wm. Jacob, Dec....268
Cotton, Alfred Cleveland, Sept..105
Couch, Asa S., Mch....413
Craft, Byron L., Dec....268
Craig, Wm. Henry, Apr....444
Curtis, Walter H., May....493

Dambach, Virginia Asher, May..493
Dellenbaugh, Feb. 359; Mch....414
Deming, Chas. C., May....493
Drake, J. B., Aug.... 4
Dundas, Thos. A., July....569

Eddy, Geo. P., Oct....161
Ely, Henry Oliver, June....533
English, Gustavus Pierce, Apr..444
Ernest, J. Glenn, Jan....314

Farrington, John M., June....533
Flood, Frank H., June....533
Frost, Henry C., Sept....106

Gamon, Frank, Apr....445
Grabenstatter, Geo. W., Feb...360
Gratwick, Martha Weare, Sept..106
Gray, Clarence V., Aug.... 41
Green, Tobias John, Dec....268
Guillaume, Clement T., July....569

Hall, Erasmus H., Jan....315
Hallett, Thos. H., May....493
Harris, Blinn A., Feb....360
Harter, Frederick J., Mch....414
Hennessey, Daniel, Jan....315

Page
Hoffmeyer, John A., Mch.......413
Horsley, Victor, Aug........... 41
Hubbard, Silas, June...........534
Hughes, Francis Mason, Jan....314

Ingraham, Lucia T. S., Feb....360

Jaeger, Wm., July.............569
Jenkins, James M., Mch........414

Kaynor, F. S., 358; corrected to

P. P., Mch...................413

Knisley, Andrew Benjamin,

Mch. ........................414

Knowlton, Chas, Benjamin,

June ........................534

Koenig, Caroline Osborn, Mch..413

Kyle, D. Braden, Nov...........211

Lane, Harry, July..............569
Langley, Wm., Sept............106
Leffingwell, E. D., Oct.........160
Leffingwell, Albert, Oct.........161
Lehmann, Joseph H., Apr......444
Lord, Matthias L., Jan.........315
Lynde, Uri Colvin, Dec.........269

McMorrow, James Aloysius,
May ..........................493
Mead, Harry, Apr..............445
Merrow, John G., Oct..........161
Miller, Adam, Dec..............268
Moffatt, John Little, Apr.......444
Moore, Richard Mott, Nov.......211
Murphy, John B., Sept.........105

Neisser, L. Albert, Sept........105

O'Hare, Thos. A., Dec.........269
Osmun, Joseph Allen Jr., Mch..413

Palmer, Stephen Elmer, Mch....414
Palmer, Wm. Everitt, Jan......314
Peck, Oxias Willard, Oct........161
Philbrick, Emmelin Pickette,
Dec. ........................268
Phillips, Hopestill R., Sept.....106
Pilcher, Paul M., Feb..........358
Pitcher, Morris O., Feb........360
Pritchard, Mahlon R., Apr......444

Ramsay, Wm., Sept............106
Ring, Wm. Grosvenor, July.....570

Schneider, Carl von Arx, Mch..414
Scouller, John Deans, June.....533
Simmons, Chas. E., June.......533
Smith, Wm. W., Mch..........414
Smither, Robert K., July........570
Spire, Clayton E., July..........570
Squire, Wm. J., June...........533
Stacey, Orrin T., Apr...........444

Page

Straight, A. M., Mch...........414

Sweeney, Richard Edward,

Feb. ........................358

Sweet, Amos L., Feb...........360

Thayer, Carnie Casander, Oct...161
Tilma, John, Feb...............360
Titus, Milton Bennett, Dec......267
Tobin, Thomas, Apr............444

Washington, Marguerite, Feb...360
Watson, Harriet Noble, June....533
Way, A. Crandall, Dec.........267
Weinbach, Christian A., Dec....268
West, Alfred C., Apr...........444
Wheeler, Claude Lamont, Feb..358
Whitbeck, John F. W., Aug.... 40
Whittleton, E. J., Dec.........268
Williams, Marcus J., May......493
Wolff, E. J., Dec...............268
Wolsley, Cardinal T., Nov.....214

### GENERAL INDEX

ACETONE, Niece Test, Abs., Dec., page 238.
Acidosis, See H-ion Concentration, Mch., page 367.
Adrenal White Line, Abs., Apr., page 450.
Adrenalin by Unusual Routes, Jan., page 318.
Alcoholism and Inebriety, T. D. Crothers, Sept., page 64.
Alcohol and Workingmen, Sept., page 88.
Alcohol and Tobacco, Statistics, Feb., page 350.
Alcohol, Deaths, Feb., page 352.
Allen Treatment in Diabetic Gangrene, Abs., Aug., page 49.
Aluminum Silicate, Alkaloid Affinities of, Abs., Dec., page 241.
Alveolar Air, Carbon Dioxid Tension, etc., La Rue Colegrove, Mch., page 367.
Ambrine, Abs., Apr., page 448.
American Balneology, See Felix von Oefele, Index of Contributors.
Amoebic Dysentery, Abs., May, page 491; Amoebae associated with dysentery bacilli, Abs., Oct., page 151; Amoebiasis, cephaline in, Abs., Feb., page 361.
Amputation, Spontaneous, Abs., Oct., page 156.
Anaemia, Symposium, June, page 506.
Anaesthesia, Local, in Surgery of

Colon and Rectum, Abs., May, page 471.
Animal Experimentation, California Laws, Apr., page 439.
Antagonism to A. M. A., Edit., June, page 513.
Anthrax, Mch., page 408; May, page 491.
Application of So-called New War-time Antiseptics in the Several Branches of Civil Life, Chas. W. Hennington, Nov., page 163.
Are We Scientific? George L. Servoss, July, page 540.
Artificial Hand, Prize for, Sept., page 89.
Asphyxiating Gases, Prophylaxis, Abs., Oct., page 149.
Attitude of the Family Physician during an Epidemic of Poliomyelitis, Irving M. Snow, Dec., page 217.
Automobile Statistics, Sept., page 93; June, page 526; Taxation, Dec., page 260; Cautions, Nov., page 191; See Highways, Jan., page 278; Radiator Solutions, Feb., page 347.
Autoplasty of Corpora Cavarnosa, Abs., Oct., page 147.
Autotherapy in Abdominal Infections, Charles H. Duncan, July, page 535.
Autotherapy in Ivy Poisoning, Abs., Jan., page 289.

BACTERIA in Commercial Bottled Waters, Nov., page 189.
Bacterial Stain, Abs., Apr., page 448.
Bacteriaemias of Agonal Period, Abs., Mch., page 375.
Bacteriology of Epidemic of Grippe, Abs., Oct., page 150.
Balneology, American, See Felix Von Oefele, Contributors' Index.
Better Doctoring for Less Money, Aug., age 30.
Biliary Affections and Pancreatitis, Chas. Gordon Heyd, June, page 495.
Bilharziosis, Cause, Abs., Aug., page 10.
Billy Sunday, Edit., Jan., page 294.
Birth and Infantile Death Statistics, Feb., page 353.
Blindness in U. S., May, page 489.
Blood Test, Abs., Jan., page 277.
Blood Pressure and Pulse on Firing Line, May, page 458.
Boy Labor, Restricting, May, page 488.
Bread, aerated, Feb., page 349.
Bread bill, Feb., page 350.
Bubbling Fountain, Mch., page 401.
Buffalo Municipal Hospital, Feb., page 351.
Buffalo Climate, Feb., page 352.
Buffalo, Univ. of Commencement, July, page 560.
Bullet in Brain, Destroying Sight and Hearing, Recovery, Evan O'Neill Kane, May, page 455.

CAESARIAN Triplets, Abs., Dec., page 229.
Canadian Nurses, Exclusion of, Sept., page 87.
Canadian Universities, Influence of War on, Jan., page 303.
Cancer, Radium Treatment, in Centenarian, Evan O'Neill Kane, Aug. page 13.
Cancer, Edit., Aug., page 19; of Cervix—Radium, Abs., Jan., page 284; Mortality, Feb., page 354; Non-Surgical vs. Surgical Treatment, Abs., (illustrated), Feb., page 361.
Carbon Dioxid Tension of Alveolar Air and Hydrogen Ion Concentration in Blood and Urine, La Rue Colegrove, Mch., page 367.
Cat License Bill, June, page 522.
Centenarians, Cancer and Radium Treatment, Evan O'Neill Kane, Aug., page 13; Notes of living and recently dead centenarians, Aug., page 34; Sept., page 90; Oct., page 141; Nov., page 191; Dec., page 262; Jan., page 307; Feb., page 351; Mch., page 404; Apr., page 439; May, page 487; June, page 522 and 528; July, page 551 and 557.
Cephaline in Amoebiasis, Abs., Feb., page 361.
Chair of Medical Ethics, Edit., Feb., page 340.
Chancre, See Syphilis.
Childless Home and Homeless Children, Oct., page 139.
Chloroma, Abs., Oct., page 160.
Chlorazene, Abs., Oct., page 158.
Chlorophyl, Abs., Sept., page 65.
Christian Science Treatment, Legal, Nov., page 194.
Chronic Intestinal Stasis from View Point of Internist, Chas. D. Aaron, Aug., page 1.
Cocaine Habit, Nov., page 191.

Cock Roaches, Abs., Feb., page 326.
Colon, Retro-peritoneal Rupture, Abs., Oct., page 148.
Concrements of Spleen, Abs., Aug., page 47.
Corpora Cavernosa, Autoplasty, Abs., Oct., page 147.
Curare Symposium, Sept., page 104.

DANYSZ Compound, No. 102, Sept., page 108; Feb., page 328.
David's (Pott's) Disease, Feb., page 141.
Deaf-Mutism, Military, Abs., Oct., page 113.
Deaths, Automobile, Feb., page 352; Infantile and Births, Feb., page 353; Cancer, Feb., page 354; of Physicians, Feb., page 347.
Degenerate Family History, Abs., Dec., page 233.
Diabetes, Gangrene, Allen Treatment in, Abs., Aug., page 49; Abs., Oct., page 125; Renal, Abs., Nov., page 172.
Diaphragmatic Hernia, Strangulated, Abs., Nov., page 172.
Dog Law, N. Y. State, June, page 522.
Doing Nicely, Editorial, Oct., page 126; Feb., page 357.
Duodenal Ulceration, Abs., Nov., page 211.
Dreaming, Edit., Jan., page 293.
Drugless Practitioners, California Law Upheld, Feb., page 351; Mch., page 402.

EASTMAN Dispensary, June, page 526.
Educational Lunch Room, Nov., page 189; Statistics, A. M. A., Sept., page 90.
Egg Albumin in Urine, Abs., Jan., page 317.
Embolism, Fat, Abs., Apr., page 447.
Emetine Causing Peripheral Neuritis, Abs., Dec., page 229.
Endorsed Hospitals, Edit., Dec., page 243.
Epilepsy, Abandonment of Post, Abs., Oct., page 149.
Erysipelas Treated with Diphtheria Antitoxin, Abs., Dec., page 238.
Ether Chloroform Mixtures, Abs., July, page 544.
Ethics, Chair of Medical, Edit., Feb., page 340.
Etiology Direct vs. Indirect Theories, Edit., Jan., page 290.
Eventration, Abs., Oct., page 148.
Examiners, National Board of Medical, Aug., page 31.
Exophthalmic Goitre, Roentgen Treatment, Abs., May, page 474.
Experience of the Summer, Edit., Oct., page 127.

FAST, Prolonged, Oct., page 142.
Fat Embolism, Abs., Apr., page 447.
Fecundity, Abs., Sept., page 63; June, page 524.
Fee-Splitting Law, July, page 553.
Flies, Destruction, Abs., Feb., page 328; Transmission of Leprosy by, Abs., Oct., page 150.
Foot Ball Casualties, Jan., page 304.

GALL Bladder Surgery, Chas. W. Hennington, Apr., page 415.
Gas Bacillus Infection, Abs., Sept., page 65.
Gases, Asphyxiating, Abs., Oct., page 149; Helmets, Agricultural Use, Nov., page 196.
Gasoline Phlegmons, Abs., Dec., page 223; Production, July, page 553.
Gastro-Intestinal Tract, Roentgen Diagnosis, Lester I. Levyn, Jan., page 271.
Gastric Ulcer and Cancer, Abs., Apr., page 448.
Generin, Abs., May, page 458.
Gonococcus and Meningococcus, Abs., Apr., page 446.
Gonorrhoea, Epidemic, Nov., page 177.
Grading of Milk Supplies, S. W. Bateson, Dec., page 239.
Grain used for Liquors, June, page 523.
Grippe Epidemic, Bacteriology, Abs., Oct., page 150.
Guthrie, Samuel, Discoverer of Chloroform, Wm. V. Ewers, May, page 459; June, page 502.

HAEMO-LYMPH, Glands, Abs., Oct., page 112.
Haemorrhoids and the General Surgeon, Rollin H. Barnes, Aug., page 11.
Haemostatic Action of Peptone in Typhoid, Abs., Mch., page 395.
Hair Snipper, Apr., page 439.

Happy Days, Those Were, Edit., Dec., page 242; Feb., page 357.
Harrison Law, Novacaine, Sept., page 91; Opium Trade, Sept., page 93; Theft of Narcotics, Sept., page 94; Decisions, Nov., page 187; Morphine and Heroine Seized, Dec., page 262; Suits, July, page 553.
Health Insurance, John D. Bonnar, May, page 472; Sept., page 88; Feb., page 348; Mch., page 389; Apr., page 439, 450; June, page 517.
Hernia, Strangulated Diaphragmatic, Abs., Nov., page 172.
Heroine, Abs., Jan., page 277.
Highways, A. L. Benedict, Jan., page 278.
Homeless Child and Childless Home, Oct., page 139.
Horse Chestnuts as Food, Abs., Oct., page 119.
Horse Serum, in Treatment of Wounds, Abs., Oct., page 149.
Hospitals, Endorsed, Edit., Dec., page 243; Economics, Edit., Dec., page 244.
Human Vivisection, Sept., page 84.
Hydrogen Ion Concentration of Blood and Urine, La Rue Colegrove, Mch., page 367.
Hydrophobia, See Rabies.
Hyperthyroidism, Roentgen Treatment, May, page 474.

INDEFINITE PAIN, Its Cause as Viewed with the Dowd Phosphatometer, B. E. Smith, Oct., page 114.
Inebriety and Alcoholism, T. D. Crothers, Sept., page 64.
Infantile Death and Birth Statistics, Feb., page 353.
Infantile Paralysis, See Poliomyelitis.
Inguino-abdominal Eventration, Abs. Oct., page 48.
Insurance Proofs and Attending Physician, Edit., Nov., page 181.
Investment, Value of Life Ins., Edit., July, page 546.
Insurance, See Health.
Intestinal Stasis, Chronic, from the Viewpoint of the Internist, Chas. D. Aaron, Aug., page 1.
Intestines and Stomach, Methods and Results in Surgery of, Geo. W. Crile, Sept., page 55.
Intrapulmonary Shrapnel, Abs., Oct., page 148.
Investments, Edit., Nov., page 178.

KIDNEY, Multiple Cystic, Wm. Stanton, Dec., page 240.
Kidney, See Renal.
Kultur, July, page 549.

LABIUM Majus, Tumor, Abs., Aug., page 21.
Lamblia Infection in England, Abs., Feb., page 336.
Law, See Harrison, Traffic, Automobile, Narcotic, Venereal, Wickes-Grant, Dog, Cat, Quack advertisement, Vaccination, Vivisection, Marriage, etc. Recent N. Y. State Laws, July, page 557.
Leaving out the Patient, Edit., Feb., page 342.
Leprosy, Transmission by Flies, Abs., Oct., page 150.
Letchworth Village, Apr., page 434.
Licensure, Requirements of Collegiate Work, Apr., page 434.
Lightning and Thunder, Edit., Oct., page 130.
Liquor Advertising, Edit., Mch., page 391.
Lithium Springs, Felix von Oefele, Apr., page 420.
Liver Abscess, Abs., Oct., page 119.
Luargol in Syphilis, Abs., Feb., page 328.
Lungs, Rupture by wind of bullet, Abs., Oct., page 148.

MAILING Poisonous Drugs, Nov., page 187.
Malaria, Man the Winter Carrier, Abs., Apr., page 446.
Marriage License Restriction, June, page 522.
Medical Brotherhood, May, page 486.
Medical Profession, Statistics, June, page 525.
Meningococcus and Gonococcus, Abs., Apr., page 446.
Menstruation, Vicarious, Abs., Jan., page 316.
Methods and Results in Surgery of the Stomach and Intestine, Geo. W. Crile, Sept., page 55.
Method of Shrinking Human Heads by Baqueta Indians, Rev. Horacio Giraloto, July, page 545.

Military Preparedness, Edit., Dec., page 242; Statistics, July, page 557.
Military Training, Compulsory and Venereal Diseases, Mch., page 401.
Militia of N. Y. State, Apr., page 437.
Mineral Oil in Peptic Ulcer, Abs., Feb., page 366.
Monstrosities, Joined Twins, Sept., page 97.
Moving, Edit., Apr., page 426.
Mycosis due to Scopulariopsis Koningi, Abs., Oct., page 150.

NARCOTIC LAW, (See Harrison), Dec., page 254.
Narcotics, Theft of, Sept., page 94; Convictions, Apr., page 436.
National Guard or ?, Edit., Mch., page 393.
National Board of Medical Examiners, Apr., page 434.. Nov., page 196; Dec., page 258; Aug., page 31.
Native Medicine Used by Pioneers, Abs., Oct., page 159.
Neosalvarsan, Smuggled and Fraudulent, Dec., page 256.
Newspaper, two-cent, Edit., Jan., page 297.
Nitrogen Metabolism during Pregnancy, Abs., Oct., page 155.
Nitrogen and Urea in Blood and Urine, Abs., Aug., page 45.
Nose, Spurs in the, John C. Warbrick, Sept., page 61.
Novacaine excluded from Narcotic Law, Sept., page 91.
Nurses, Exclusion of Canadian, Sept., page 87.

OCCULT Blood Test, Abs., Sept., page 60.
Ochronosis and Melanuria, Abs., Aug., page 12.
Omentum, Bullets extracted from, Abs., Oct., page 148.
Ophiophobia, Fatal, Apr., page 436.
Opium Trade, Sept., page 93.
Opportunity of the Medical Profession, Edit., June, page 514.
Ossification, Absence of, Abs., Oct., page 119.

PANCREATITIS and Biliary Affections, Chas. Gordon Heyd, June, page 495.
Paratyphoid (See Typhoid) Inoculation with grave phenomena, Abs., Nov., page 210; Carriers, Dec., page 259.
Parturient Mortality, June, page 522.
Passing Race (Colonial Americans), Abs., Feb., page 364.
Pathogeny, A Priori Reasoning as to, Edit., May, page 479.
Pensions for Physicians, Edit., Sept., page 71.
Peptic Ulcer, Mineral Oil, Abs., Feb., page 366.
Periodicals, Statistics of, Oct., page 138.
Peripheral Neuritis following Emetine, Abs., Dec., page 229.
Pharynx, The, John C. Warbrick, Feb., page 327.
Phlegmons, Gasoline, Abs., Dec., page 223.
Phylacogen Treatment of Rheumatism, H. C. Lapp, Sept., page 60.
Physical Condition of Volunteers, Nov., page 191.
Physicians, Deaths of, Feb., page 347.
Pituitary Extract, See Tethalin.
Pleural Cavity, Bullet free in, Abs., Oct., page 148.
Poisonous Drugs, Mailing, Nov., Page 187.
Poison Ivy Autotherapy, Abs., Jan., page 289.
Poliomyelitis, Epidemic, Sept., page 92; Aug., page 35; Edit., Sept., page 76; Sept., page 88; Dec., page 262; Routes of Travel, Edit., Oct., page 131; Oct., page 142 and 145; Bates Bill, Nov., page 188; After care, Mch., page 400; Nov., page 194; Intestinal Lesions, Nov., page 195; Nov., page 195, 196 and 200; Attitude of Family Physician during Epidemic, Irving M. Snow, Dec., page 217; Differential Diagnosis and Treatment, E. A. Sharp, Dec., page 224; Edward Clark, Dec., page 230; Mortality, Jan., page 305; Jan., page 307; June, page 501; Clinic, June, page 528; July, page 553.
Polygamy and polyandry, Abs., Jan., page 297.
Pott's Disease, See David's.
Pregnancy, Skin Reaction, Abs., Apr., page 424.

Preparedness, Military, Edit., Dec., page 242; Medical Military, Edit., Feb., page 352.
Prize for Artificial Hand, Sept., page 89; McIntire, Mch., page 404; Alexander Graham Bell Grosvenor, Mch., page 405.
Prognostic Value and Clinical Application of Studies in Retention, Richard M. De Niord, Feb., page 319.
Prohibition, Opposition to, Feb., page 348.
Projectiles, Saturnism from, Abs., Oct., page 148.
Public School Registration, Buffalo, Dec., page 259.
Pulse and Blood Pressure on Firing Line, Abs., May, page 458.

QUACK Advertisements, June, page 523.
Quintuplets, Abs., June, page 534.

RABIES, State Quarantine, Sept., page 87; Human in California, Abs., Sept., page 63; Jan., page 304; Case in a Boy 9 years old, Francis E. Fronczak, Feb., page 337; Mch., page 404; June, page 528.
Radial Artery, Double, Abs., July, page 544.
Radiculitis, Abs., Aug., page 46.
Radium, Case History of Centenarian—Cancer, Evan O'Neill Kane, Aug., page 13; in Cancer of Cervix, Abs., Jan., page 284.
Red Cross, Apr., page 425; July, page 558.
Renal Diabetes, Abs., Oct., page 125.
Renal Lesions, Clinical and Pathologic Diagnosis, Abs., Jan., page 316.
Renaissance of Profession, Edit., Aug., page 16.
Retention, Studies in, Richard N. De Niord, Feb., page 319.
Retroperitoneal Rupture of Colon, Abs., Oct., page 148.
Rheumatism, Phylacogen Treatment, H. C. Lapp, Sept., page 66; Bacterial Studies, Abs., July, page 572.
Rockefeller Foundation Appropriations, May, page 491; June, page 526.
Roentgen Diagnosis of Gastro-intestinal Tract, Lester I. Levyn, Jan., page 271; Treatment of Exophthalmic Goitre and Hyperthyroidism, Abs., May, page 474; Oct., page 151.
Routes of Travel, Edit., Oct., page 131.
Rupture of Both Lungs by Wind of Bullet, Abs., Oct., page 148.

SATURNISM from Projectiles, Abs., Oct., page 148.
Scientific Researches into the Causes of Alcoholism, T. D. Crothers, Sept., page 64.
School Registration, Buffalo, Dec., page 259.
Sedentary Occupations, Edit., May, page 475.
Shell Bullet in Omentum and Movable in Hernial Sac, Abs., Apr., page 419; Delivery Through Shell Wound, Abs., July, page 571.
Shrinking Human Heads, Horacio Giraloto, July, page 545.
Six-Year Medical Course, Jan., page 304.
Small Hole for the Kitten, Edit., Apr., page 427.
Social Importance of Vital Statistics, Edit., Feb., page 339.
Sorehead, The, Edit., Aug., page 20.
Spleen, Concrements of, Aug., page 47.
Spontaneous Amputation, Abs., Oct., page 156.
Sprue, Abs., Jan., page 318.
Spurs in the Nose, John C. Warbrick, Sept., page 61.
Standard Oil Earnings, June, page 523.
Stasis, Chronic Intestinal, Chas. D. Aaron, Aug., page 1.
State Board Examinations, May, page 487.
Statistics of Medical Profession, June, page 525. (See Birth, Death, Automobile, etc.)
Stomach and Intestines, Methods and Results in Surgery of, Geo. W. Crile, Sept., page 55.
Streptococcus Mucosus Capsulatus, its Relation to Otology, Homer A. Trotter, Nov., page 169.
Sublingual Medication, Jan., page 289.
Summer, Experiences of the, Edit., Jan., page 294.
Sunday, Billy, Edit., Jan., page 294.
Sunstroke, Sept., page 93.

Superfoetation, Abs., Sept., page 65.
Sword Swallower, Death of, Abs., Feb., page 366.
Syphilis, Cured by one dose of Salvarsan, Reinfection two years later, Chas. W. Bethune, Oct., page 113; Chancre of Conjunctiva, Abs., Apr., page 450; Oct., page 154; of Stomach, Abs., Oct., page 154; Luargol, Abs., Feb., page 328; Inherited from paternal great grandfather, Mch., page 373; Pulmonary, Abs., Apr., page 447; Oct., page 152.

TAPE WORM, Wandering, Abs., Jan., page 284.
Telephone Merger, Oct., page 139.
Tetanus cured by massive doses of serum, Abs., Oct., page 150; Localized, Abs., Nov., page 209.
Tethalin (Pituitary Extract), Abs., Feb., page 347.
Those Were Happy Days, Edit., Dec., page 242.
Thunder and Lightning, Edit., Oct., page 130.
Thymus Disorders in Adults, Abs., Sept., page 63.
Tobacco and Liquor Statistics, Feb. page 350.
Traffic, Proposed Uniform Legislation, Dec., page 252; Law, July, page 555.
Trapezoid, Fracture, Abs., June, page 505.
Triplets, Caesarian, Abs., Dec., page 229; 3 times and twins, Abs., Sept., page 63.
Trypsin in Cancer, Abs., Apr., page 449.
Tuberculosis, Wassermann Reaction, Abs., Feb., page 338; Decline, Mch., page 401; Hospitals, Apr., page 435; July, page 553; Clinics, Buffalo, Apr., page 438; Open Air Camp, Buffalo, June, page 524; Much-Weiss Granules, Abs., Apr., page 447; Concentrating Bacilli in Urine, Abs., Apr., page 424; Conjugal, July, page 544.
Buffalo, June, page 524; Much-Weiss Granules, Abs., Apr., page 447; Concentrating Bacilli in Urine, Abs., Apr., page 424.
Twilight Sleep, Apr., page 424; Sept., page 97.
Twins, Death of old, Feb., page 352; Joined, Abs., Sept., page 97.
Two-cent Newspaper, Edit., Jan., page 297.
Typhoid, Test in Nurses and Physicians, Abs., Apr., page 449; Carrier, Feb., page 352; Peptone in Haemorrhage, Mch., page 395; Inoculation, Injunction Refused, Mch., page 400; Reduction in Toronto, Sept., page 96; Reactions in Testicle, etc., Abs., Oct., page 149; Diplococcaemia, Abs., Oct., page 150; Atypic, Abs., Oct., page 156; Typhoid and Paratyphoid Bacilli in Vomit, Abs., Nov., page 209; Proportionate Influence of Typhoid and Paratyphoid as Influenced by Vaccination, Abs., Nov., page 209. (See Paratyphoid).
Tyrocoxicon, Abs., June, page 534.
U. S. ARMY Medical Corps Increase, Dec., page 257; Navy Corps Increase, Dec., page 256.
Unsafe Inference, Edit., Sept., page 74.
Urea and Nitrogen of Blood and Urine, Abs., Aug., page 45.
Urea, Toxic Effects, Abs., Apr., page 445.
Urine, Egg Albumin in, Abs., Jan., page 317.

VACCINATION Bill, N. Y. State, June, page 523. (See Variola, Typhoid, Paratyphoid, Inoculation, etc.)
Variolar, July, page 550.
Vena Cava Sup., Aneurismal Obstruction, July, page 572.
Venereal Instruction Required by Law, N. Y. State, June, page 525.
Vincent's Angina, Sequellae, Abs., Nov., page 210.
Vinum Cardui Suit, Aug., page 33.
Vision of the School Child, Abs., Dec., page 223.
Vital Statistics, Social Importance of, Edit., Feb., page 339; Possible Fallacy in, Edit., Apr., page 425; Indians, July, page 550.
Vitamines, Abs., Aug., page 49.
Vivisection, California Law, Apr., page 439; Human, Sept., page 84; License Denied, July, page 554.
Volunteers, Physical Condition, Nov., page 191.

WAR LOSSES, Dec., page 256; among British Surgeons, May, page 488; Nov., page 190; Influence on Canadian Universities, Jan., page 303; Canadian Casualties, Jan., page 303; June, page 526; Statistics, Sept., page 93 and 96; Oct., page 139; July, page 554; German Wounded, Mch., page 404; Thoughts on, Edit., Apr., page 450; Duties of Medical Profession, Edit., May, page 476; Recent Graduates Available, May, page 487; Health of German Army, May, page 488; Medico-Military Materiel, May, page 489; Preparedness of N. Y. State, Health Dept., May, page 490; Children in War Time, May, page 490; Basis of Compensation of Medical Officers, Edit., June, page 516; R. R. cars required for Military Purposes, June, page 523; Military Potentiality, June, page 524; Protection of Physicians on Military Duty, June, page 528; English Authority to Medical Officers, July, page 549.

Warts, Abs., Oct., page 154.

Wealth, National, Apr., page 433.

White Slavery, Dec., page 266.

Wickes-Grant Bill, N. Y. State, Apr., page 436.

Wind of Bullet—Rupture of Both Lungs, Abs., Oct., page 148.

With the 74th Regiment on the Texan Border, H. H. Glosser, Oct., page 120.

Women Physicians, Priority, Oct., page 141.

Workingmen and Alcohol, Sept., page 88.

X-RAYS, See Roentgen.

